# New Liquid Gas Apparatus and Dental Cabinet

**Published:** 1883-08

**Authors:** 


### NEW LIQUID GAS APPARATUS AND DENTAL CABINET.

Dr. B. M. Wilkerson, late editor of this journal, the inventor of the Wilkerson chair and many other appliances largely used in dentistry, will exhibit at the coming meeting of the American Dental Association at Niagara Falls, a new liquid gas apparatus, and an operating cabinet, both of which materially differ from anything now in use, and which we think will be received with favor by the profession. As they are so soon to be submitted to the critical inspection of the best judges, we shall not here attempt any explanation or description of them.

